# Sudden onset dyspnea caused by Bochdalek diaphragmatic hernia in an adult: a case report

**DOI:** 10.1186/s13256-021-02907-1

**Published:** 2021-08-05

**Authors:** Luis Alfonso Machado Contreras, Stephanie Paola Retamoso Díaz, Gerardo Valencia, Luis Carlos Morales, María José Herrera Bedoya

**Affiliations:** 1 Medical Student, Universidad del Norte, Barranquilla, Colombia; 2 Emergency Doctor, Fundación Hospital Universidad del Norte, Barranquilla, Colombia; 3 Department of Medicine, Universidad del Norte, Barranquilla, Colombia; 4 Department of Pathology, Fundación Hospital Universidad del Norte, Barranquilla, Colombia

**Keywords:** Bochdalek diaphragmatic hernia, Respiratory failure, Stomach, Lungs, Necropsy

## Abstract

**Background:**

Bochdalek diaphragmatic hernia is a developmental defect of the posterolateral portion of the diaphragm. This defect may allow abdominal contents to abnormally occupy the thoracic cavity, resulting in most cases in the compression of the developing lungs. Signs are typically shown during early childhood since the defect is usually present during development. In exceptional cases, however, Bochdalek diaphragmatic hernia can be observed in asymptomatic adult patients, or in those whose initial diagnosis may include common respiratory pathologies such as asthma.

**Case presentation:**

Here we describe the case of a 31-year-old Mestizo female patient admitted to the emergency room owing to sudden onset of pain in the left hypochondrium and in epigastrium, as well as signs of respiratory distress. Soon after admission, the patient entered cardiorespiratory arrest, and advanced cardiac life support was provided for 45 minutes without success. The patient was declared dead 1 hour 40 minutes after admission. Clinical autopsy concluded that cause of death was respiratory failure as a complication of a previously undiagnosed Bochdalek diaphragmatic hernia.

**Conclusions:**

We report an exceptional case of Bochdalek diaphragmatic hernia as the cause of rapid-onset respiratory failure and death in an adult. Unfortunately, due to its unusual presentation, Bochdalek diaphragmatic hernia is rarely considered among the list of differential diagnoses when admitting an adult patient with respiratory symptoms. By reporting this case, we encourage the medical community and trainees to consider diaphragmatic defects when approaching a patient with sudden onset of abdominal pain with concomitant respiratory symptoms.

## Background

Posterolateral or Bochdalek hernia (BH) is a defect in the embryonic development of the diaphragm. It is characterized by a complete or partial absence of the posterior or lateral diaphragmatic muscle, which is often accompanied by protrusion of the stomach, intestines, liver, and/or spleen into the thoracic cavity [1]. BH is the most common and clinically relevant type of congenital diaphragmatic hernia, accounting for approximately 70% of the documented cases [2].

Prevalence of BH is estimated at 2–2.5/7000 live births, affecting both sexes equally. BH is extremely rare in adults, accounting for only 0.17–6.0% of all diaphragmatic hernias diagnosed during adulthood [3]. In BH, the defect is found on the left side in 85% of cases, on the right side in approximately 10%, and bilaterally in nearly 5% of cases [1, 2]. Respiratory symptoms are the hallmark of BH, although gastrointestinal symptoms can also be found. Episodes of respiratory distress secondary to pulmonary hypoplasia are very common, especially when the defect is present during early childhood or when its size is significant [3]. Adult diaphragmatic hernias commonly remain undiagnosed, and are mostly detected as an incidental finding in imaging studies [4].

Diagnosis of late congenital diaphragmatic hernia is usually confirmed by barium x-ray studies or by computed axial tomography [4]. In the case reported here, it was not possible to perform any imaging study because of the rapid clinical worsening of the patient, and BH was only confirmed after clinical autopsy.

The pathogenesis of BH is not fully elucidated yet, although 218 genes have been linked to diaphragmatic structural defects. Of those, 54 genes (such as CRABP1, CRABP2, LRAT, RBP1, RBP2, and RBP5) are involved in retinoic acid metabolism or its signaling pathways. This suggests that retinoic acid signaling could be an important regulator of embryogenesis of the diaphragm, and that defects in this pathway or its downstream targets could contribute to the development of BH [2]. Animal studies suggest that mutations in the GATA4 gene, associated with the formation of diaphragmatic connective tissue, may play an important role in the development of congenital diaphragmatic hernias. It has been hypothesized that mutations in GATA4 would cause weaker connective tissue, facilitating herniation of abdominal content into the thoracic cavity [5]. Thus, we can conclude that BH may have a multifactorial origin, since the loss of a GATA4 allele alone is not sufficient to cause diaphragmatic defects, suggesting that the development of BH may require the interaction of various genetic or environmental factors.

## Case presentation

A 31-year-old Mestizo woman was admitted to the emergency room of Hospital de la Universidad del Norte. The patient and relatives confirmed she arrived from a different hospital where she remained for approximately 5 hours before voluntarily checking out against medical advice. When admitted to our ER, the patient’s chief complaint was acute pain in left hypochondrium and epigastrium, radiating to her back. Vitals at arrival were within normal ranges, but the patient showed signs of mild respiratory distress. Thorax and abdominal auscultation at arrival were normal, according to medical records. There is no record of oxygen saturation levels. Patient also complained about nausea and reported three emetic episodes. No history of abdominal or thoracic trauma was reported by the patient. The patient denied past surgical history or recent use of drugs. However, the patient and her relatives reported a previous diagnosis of asthma with low bronchodilator responsiveness. After triage, the patient reported an increase in pain and showed paleness, tachycardia, tachypnea, abdominal distension, and timpanism. At this moment, an acute abdomen was diagnosed, and the patient was prepared for hospitalization. Pancreatitis and perforated hollow viscus were considered as differential diagnoses. Laboratory analyses requested included hemogram and blood levels of amylase, total and direct bilirubin, plasma chloride, lipase, blood urea nitrogen (BUN), liver enzymes [aspartate transaminase (ALT) and alanine transaminase (AST)], and creatinine. Imaging studies requested included abdominal ultrasound and x-rays. Additionally, intravenous saline, hyoscine butylbromide (20 mg, intravenous), tramadol hydrochloride (50 mg, intravenous), and oxygen by nasal cannula (low flow) were prescribed. During catheterization, the patient entered cardiorespiratory arrest (asystole); therefore, no blood sample was collected for the laboratory analyses requested, and no imaging analysis was possible. Advanced cardiac life support (ACLS) was initiated, and endotracheal intubation was performed. Ventilation and chest compressions were performed according to protocol for 45 minutes. An ultrasound scan was performed because of the high resistance encountered while inserting the endotracheal tube, the marked abdominal distension, and the presence of subcutaneous emphysema in the neck and inguinal regions that developed during resuscitation. Tension pneumothorax was ruled out, but evidence of a large volume of intraintestinal gas was found.

The declaration of death was done 1 hour and 40 minutes after her admission to our hospital. Patients’ relatives were informed, who requested a necropsy to determine the cause of death.

### Necropsy and macropathology reports

**Fig. 1:**
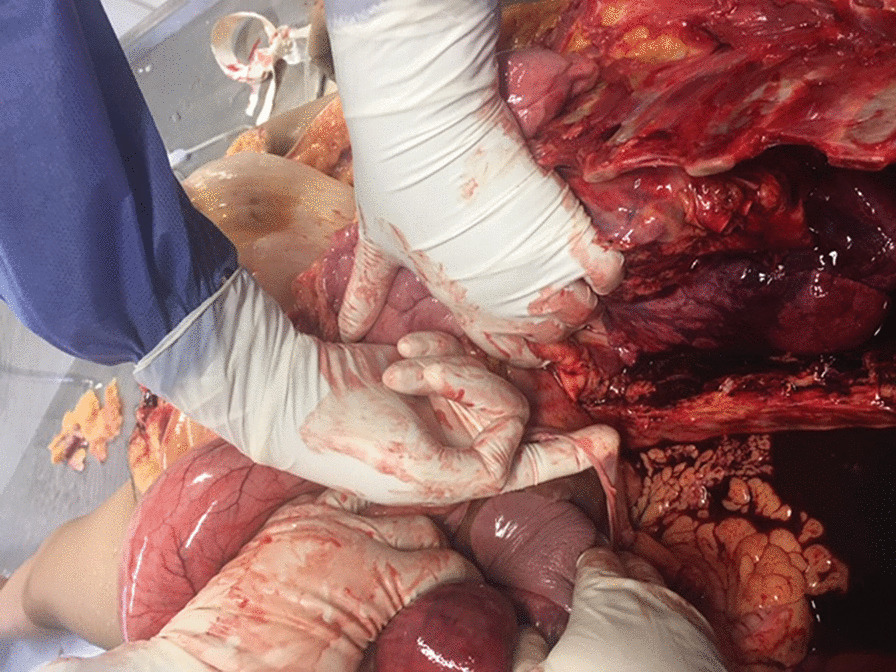
A diaphragmatic defect is observed. The region of the diaphragm where the defect was present was almost translucent because of the absence of muscular layer. Diaphragm surrounding the defect is composed almost exclusively of fibrous tissue. The left lung is collapsed because of the presence of abdominal content

**Fig. 2:**
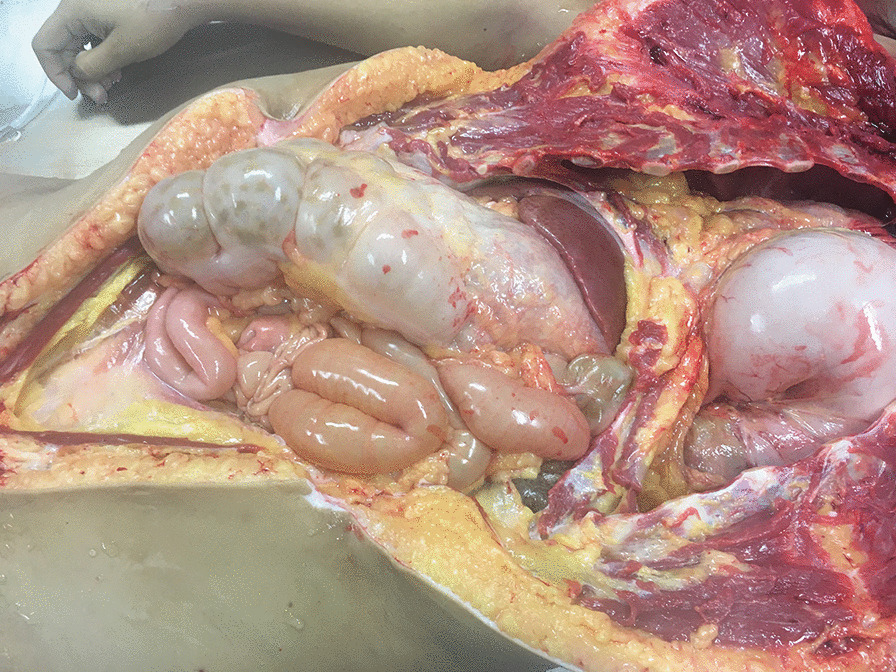
Stomach and the first portion of the duodenum is found within the thorax, herniated through the diaphragmatic defect. Dilation of the abdominal viscera was also observed

A clinical autopsy was performed in the pathology service of Hospital de la Universidad del Norte. An external description and general characterization of the body was carried out, followed by an internal examination. Photographic evidence was collected at each step. At first sight, it was obvious to the examiner that abdominal viscera were present within the thoracic cavity, predominantly in the left hemithorax. Fibrous adhesions between pleura, thoracic wall, and the viscera were observed. Abdominal viscera, mostly stomach and a portion of duodenum, were found to protrude to the thorax through a diaphragmatic hernia of approximately 10 cm in diameter located on the left side of the diaphragm (Fig. 1). Stomach within the thorax showed extensive areas of ecchymosis. Left lung was found collapsed because of pressure exerted by the stomach, and a clear deviation of mediastinum to the right was observed (Fig. 2). In addition, a sternal fracture was found, most likely caused by resuscitation. Although we did not find costal fractures, it is likely that trauma secondary to chest compressions could explain the subcutaneous emphysema described above.

In the peritoneal cavity, there was distension of the duodenum, jejunum, ileum, colon, and cecum with edematous and pale walls. Multiple fibrous adhesions between intestinal loops were also observed. Incidentally, it was also found that the patient had a bilateral duplicated collecting system. No evidence of intestinal perforation was found. Due to the localization of the diaphragmatic hernia, a diagnosis of BH was considered. Tissue samples of various organs as well as from different segments of the diaphragm, including the vicinity of the hernia, were collected for further histopathologic analyses.

### Histopathology report

Tissue samples of less than 1 cm^2^ in volume were obtained, with subsequent preservation in 10% neutral buffered formalin at room temperature. Tissue samples were processed in a Donatello automatic tissue processor (DiaPath, Italy), and sequential 4-μm-thick cuts were done in a HistoCore Arcadia H semiautomated microtome (Leica, Germany), followed by routine staining with hematoxylin and eosin for subsequent histopathological analysis and photographic documentation.

**Fig. 3:**
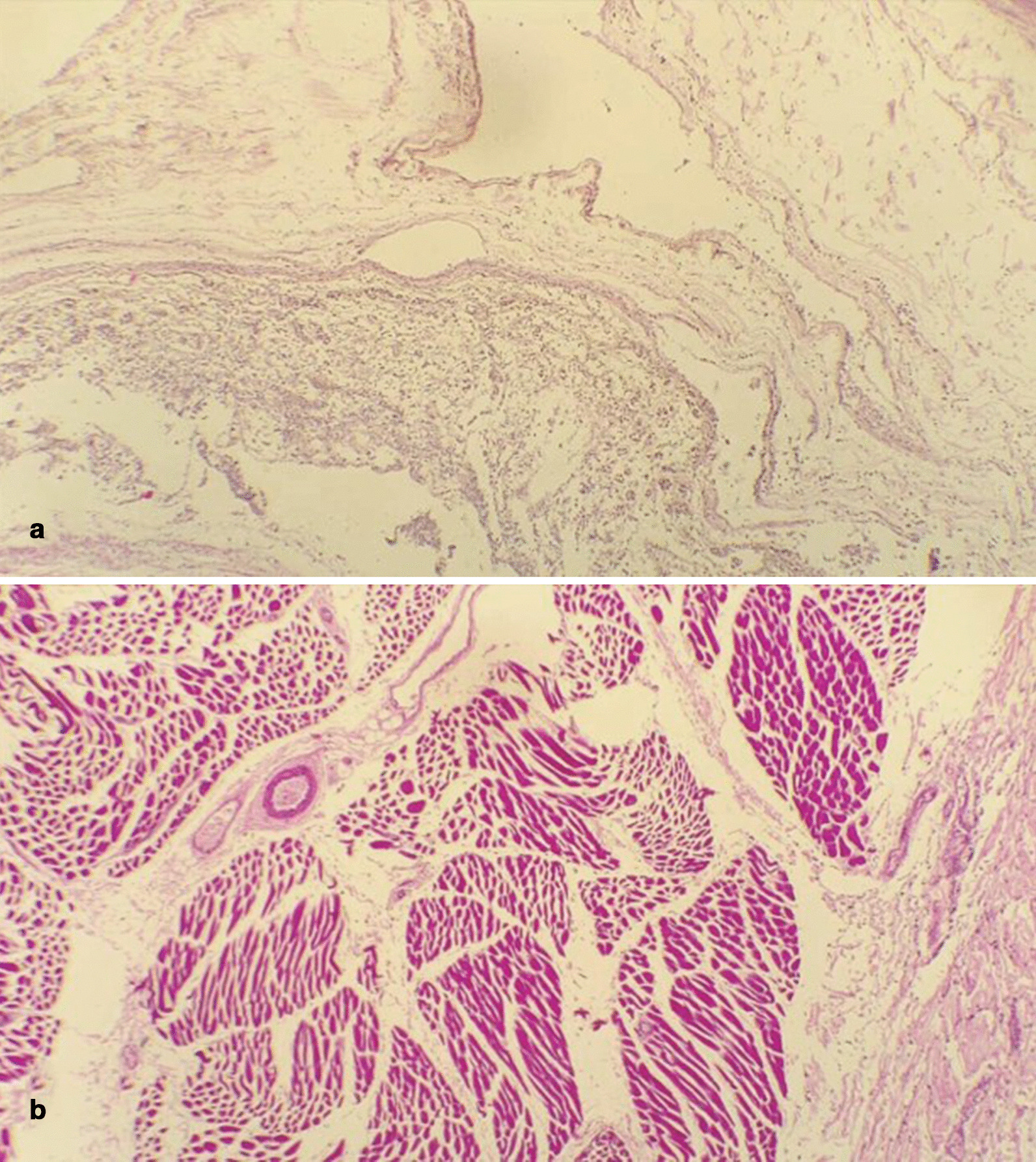
**a** Histopathology sample obtained from the vicinity of the diaphragmatic defect showing the absence of muscular tissue, which was replaced by fibrous tissue. **b** For comparison, histopathology sample obtained from the right side of the diaphragm. It is possible to observe the conserved muscular layer. Slides were stained with hematoxylin–eosin; microphotographs are shown at 10× magnification

The samples of the diaphragm collected from the vicinity of the hernia contained abundant fibrous connective tissue, showing congestion and edema. It was possible to observe mixed inflammatory infiltrate where polymorphonuclear lymphocytes and neutrophil were identified among the scattered areas of fibrosis. Notably, the amount of muscle fibers within these samples was abnormally low or completely absent (Fig. 3a). Patients’ normal diaphragmatic tissue samples were taken for comparison purposes. These diaphragmatic muscle fibers showed normal histological characteristics, although mild congestion and edema were also observed in these areas (Fig. 3b).

In the left lung (collapsed by the abdominal content), congestive pulmonary parenchyma with areas of rare focal anthracosis and hemorrhage was observed. Large atelectasis and enlarged alveolar septa with chronic inflammatory infiltrate and fibrotic zones were present. The right lung showed areas of emphysematous appearance with marked congestion as well as the presence of chronic inflammatory infiltrate and signs of hemorrhage in the alveolar septa. The examiner concluded that, given the macro- and microscopic findings, the patient was suffering from previously undiagnosed BH.

## Discussion and conclusions

Bochdalek diaphragmatic hernia was first described in 1848, and it was not until 54 years later that a first successful surgical approach for BH was published [2]. Nowadays, BH is the most frequent congenital diaphragmatic hernia reported in infants. In most of the cases, the defect is found on the left hemidiaphragm (80–85% of cases) [5], and it is correlated with higher mortality risk when a prenatal diagnosis cannot be made. BH differs from Morgagni hernia in that the latter has a lower incidence and the defect is usually located on the anterior wall [6].

As a congenital defect, symptoms are usually present at an early age, mostly due to pulmonary hypoplasia, surfactant dysfunction, or hypoplasia of cardiac structures, among other deformations caused by the herniation of abdominal content to the thoracic cavity. Previous reports have shown that less than 10% of total cases of BH are found in adults [7], and of these, up to 14% may remain virtually asymptomatic until detected incidentally [5]. In some adult BH patients, the presence of transient respiratory symptoms could lead to misdiagnosis of asthma, as we believe was the case of the patient in this report.

Here we describe a previously undiagnosed 31-year-old patient who was admitted to the emergency room because of sudden onset abdominal pain associated with respiratory distress. Unfortunately, the condition of the patient worsened shortly after admission to our medical center, entering cardiorespiratory arrest. Although ultrasound could be performed during resuscitation, ruling out pneumothorax and/or pleural effusion, it was not possible to get detailed images of the contents of the thorax or abdomen because of gas-filled small-bowel loops, which would explain the abdominal distension evidenced since admission. Since the patient quickly deteriorated, it was not possible to obtain or analyze blood samples, nor perform imaging studies that could have potentially led to the correct diagnosis of the patient.

According to the patient’s relatives, she had previously complained about symptoms that resembled asthma attacks and did not respond to medications. This may be explained by the congenital diaphragmatic defect found during necropsy, that may have allowed her stomach to ascend into her thorax and subsequently return into the abdominal cavity, leading to a transient compression of the left lung and causing symptoms that may have resembled an asthma attack. Additionally, the multiple fibrous adherences found in abdomen and thorax could be evidence of long-term and chronic inflammation of the intestinal and pleural serosa, suggesting that the defect had been allowing abdominal content into the thorax in the past.

We believe that the event that led the patient to the emergency room was the stomach being incarcerated and, hence, not being able to return to the abdominal cavity. This generated a constant compression of the left lung, causing the patient to show shortness of breath, in turn leading to an increase in her respiratory rate, promoting aerophagia, increasing the space that the stomach was occupying in the thorax, and causing a vicious cycle of further compression of the left lung and displacement of the mediastinum to the right side, causing a deviation of the trachea, which possibly explains the resistance encountered during the intubation procedure. We hypothesize that the growing compression of the heart caused a decrease in the ejection fraction and an increase in retrograde pressure. Thus, the blood coming through the pulmonary veins to the left atrium began to accumulate in the pulmonary vessels, increasing hydrostatic pressure in said vessels, which subsequently led to edema and to a further decrease in the patient’s gas exchange capacity. Interestingly, despite the abdominal distention and the subcutaneous emphysema, the autopsy did not report rupture of the esophagus or any other hollow viscera. We hypothesize that, perhaps, rupture of lung/pleural tissue could be the cause of it, given the sternal fracture found during autopsy.

In addition to the incarceration of the stomach within the thoracic cavity, we also found dilation of the abdominal viscera, and although the incarceration of the stomach could not fully explain abdominal distention, we believe that it could have made it difficult to lower the diaphragm and also reduced the venous return to the heart, further decreasing the preload and ejection fraction, leading to systemic hypoxia and, subsequently, to multiorgan failure. It is also possible that the previously formed abdominal adhesions could have caused an intestinal obstruction, increasing the intraabdominal pressure, which could have favored herniation. Although this hypothesis could not be ruled out, we consider this to be unlikely because of the rapid worsening of the patient. Understanding the occurrence of all these events and how they developed could help to explain the rapid pace at which the patient’s symptoms progressed and how they caused her death in a very short time.

In conclusion, the patient’s cause of death could be explained by the combination of an increase in intraabdominal pressure secondary to dilation of the viscera, and mediastinal syndrome secondary to the presence of the stomach in the chest. To our knowledge, few other cases in which BH is linked to sudden dyspnea in previously undiagnosed adult patients have been reported [8], but in this particular case, compared with previously reported cases, the patient’s condition worsened quickly, resulting in her death. Therefore, diagnosis was possible only after postmortem examination. Before death, the patient was not able to provide any indication of previous imaging studies that could have suggested to the authors the presence of the hernia. It was also very unfortunate that we could not access the patient’s previous medical records that could have helped to clarify the diagnosis of the patient. We believe that BH is rarely considered as a potential cause of dyspnea in adults. This report may help doctors and trainees to consider diaphragmatic hernias as a potential cause of respiratory failure in adult patients.

## Data Availability

Data sharing is not applicable to this article as no datasets were generated or analyzed during the current study.

## Abbreviations

ACLS: Advanced cardiac life support
AST/ALT: Aspartate transaminase/alanine transaminase
BH: Bochdalek hernia
BUN: Blood urea nitrogen
cm: Centimeters
CRABP1: Cellular retinoic acid-binding protein 1
CRABP2: Cellular retinoic acid-binding protein 2
GATA4: GATA-binding protein 4
LRAT: Lecithin retinol acyltransferase
RBP1: Retinol-binding protein 1
RBP2: Retinol-binding protein 2
RBP5: Retinol-binding protein 5
